# Engineering *Pseudomonas putida* for production of 3-hydroxyacids using hybrid type I polyketide synthases

**DOI:** 10.1016/j.mec.2025.e00261

**Published:** 2025-04-02

**Authors:** Matthias Schmidt, Aaron A. Vilchez, Namil Lee, Leah S. Keiser, Allison N. Pearson, Mitchell G. Thompson, Yolanda Zhu, Robert W. Haushalter, Adam M. Deutschbauer, Satoshi Yuzawa, Lars M. Blank, Jay D. Keasling

**Affiliations:** a Joint BioEnergy Institute, 5885 Hollis Street, Emeryville, CA 94608, USA; b Biological Systems & Engineering Division, Lawrence Berkeley National Laboratory, Berkeley, CA, 94720, USA; c Institute of Applied Microbiology (iAMB), Aachen Biology and Biotechnology (ABBt), RWTH Aachen University, 52062 Aachen, Germany; d California Institute for Quantitative Biosciences (QB3), University of California, Berkeley, CA, 94720, USA; e Department of Plant and Microbial Biology, University of California, Berkeley, CA, 94720, USA; f Joint Program in Bioengineering, University of California, Berkeley/San Francisco, CA, 94720, USA; g Department of Chemistry, University of California, Berkeley, CA, 94720, USA; h Environmental Genomics and Systems Biology Division, Lawrence Berkeley National Laboratory, Berkeley, CA, 94720, USA; i Department of Chemical and Biomolecular Engineering, University of California, Berkeley, CA, 94720, USA; j Systems Biology Program, Graduate School of Media and Governance, Keio University, Fujisawa, Kanagawa, 252-0882, Japan; k Institute for Advanced Biosciences, Keio University, Tsuruoka, Yamagata, 997-0017, Japan; l The Novo Nordisk Foundation Center for Biosustainability, Technical University of Denmark, 2800 Kgs. Lyngby, Denmark

**Keywords:** Pseudomonas putida, Polyketide synthase engineering, 3-Hydroxyacid production, Transposon sequencing, Protease degradation tag

## Abstract

Engineered type I polyketide synthases (T1PKSs) are a potentially transformative platform for the biosynthesis of small molecules. Due to their modular nature, T1PKSs can be rationally designed to produce a wide range of bulk or specialty chemicals. While heterologous PKS expression is best studied in microbes of the genus *Streptomyces*, recent studies have focused on the exploration of non-native PKS hosts. The biotechnological production of chemicals in fast growing and industrial relevant hosts has numerous economic and logistic advantages. With its native ability to utilize alternative feedstocks, *Pseudomonas putida* has emerged as a promising workhorse for the sustainable production of small molecules. Here, we outline the assessment of *P. putida* as a host for the expression of engineered T1PKSs and production of 3-hydroxyacids. After establishing the functional expression of an engineered T1PKS, we successfully expanded and increased the pool of available acyl-CoAs needed for the synthesis of polyketides using transposon sequencing and protein degradation tagging. This work demonstrates the potential of T1PKSs in *P. putida* as a production platform for the sustainable biosynthesis of unnatural polyketides.

## Highlights

• General assessment of a non-native polyketide synthase (PKS) host.
• CoA pool expansion of *Pseudomonas putida* to diversify PKS products.
• Functional genomics to improve acyl-CoA supply for polyketide production.
• Further engineered initial PKS design by acyl transferase (AT) swapping.
• 3-hydroxyacids production with varying chain lengths and branching patterns.

## 1 Introduction

The chemical industry is a major contributor to global CO_2_ emissions, with the production of chemical building blocks still heavily reliant on fossil resources (Gabrielli et al., 2023). One limitation to the replacement of fossil resources with renewable resources (e.g., lignocellulosic biomass, sugars) is the lack of highly flexible biosynthetic pathways to produce the chemicals now derived from petroleum. Significant advances in enzyme design and directed evolution have greatly expanded the range of molecules that can be synthesized using biological chemistry, and the resulting engineered pathways tend to be bespoke for each molecular target (Li et al., 2000).

A highly versatile approach for the biosynthesis of customized small molecules is the use of engineered type I polyketide synthases (T1PKSs), especially when performed in industrially relevant microbes. Over the years, the modularity of PKSs have made them a compelling target for engineering, allowing for the synthesis of a diverse range of unnatural products (Donadio et al., 1991; Englund et al., 2023b; Hagen et al., 2016; Yuzawa et al., 2017, 2018). Because T1PKSs are predominantly found in actinomycetes, their expression in other hosts remains in its infancy (Kumpfmüller et al., 2016; Pfeifer and Khosla, 2001). The yields are frequently suboptimal, and given the vast diversity of polyketides, formulating a one-size-fits-all strategy for enhancement proves challenging (Chai et al., 2012; Dudnik et al., 2013; Gross et al., 2006a, 2006b; Loeschcke and Thies, 2015; Wenzel et al., 2005). Given the substantial size and intricacy of PKSs, their expression is likely to be a significant bottleneck. While this constraint was partially eased by codon optimization, another pivotal aspect to consider is the availability of precursors (Schmidt et al., 2023). Consequently, refining both the host's metabolic pathways and the composition of the culture medium appear as key optimization strategies.

*Pseudomonas putida* has emerged as a promising expression host for PKSs (Loeschcke and Thies, 2015). Due to its omnivorous characteristics, the use of *P. putida* as a production platform often requires an intense study of the metabolic network and the elimination of catabolic pathways. In this context, RB-TnSeq has become a powerful tool in *P. putida* (Incha et al., 2020; Rand et al., 2017; Schmidt et al., 2022; Thompson et al., 2020; Wetmore et al., 2015). This technique merges the generation of transposon mutant libraries from TnSeq with the use of uniquely barcoded single mutants from BarSeq (Smith et al., 2009; van Opijnen et al., 2009). After generating a transposon mutant library and assigning each transposon insertion site to a unique DNA barcode, the mutant pool is cultivated under selective conditions. By quantifying the DNA barcodes before and after cultivation, potential changes in the abundance of genetic phenotypes can be detected, and gene essentiality can be derived. These data can then be used to make more informed decisions about genetic host modifications (Thompson et al., 2019b).

To demonstrate the capabilities of *P. putida* KT2440 as a foundation for engineered T1PKSs, we chose the lipomycin synthase of *Streptomyces aureofaciens* Tü117 (LipPKS) to produce industrially relevant 3-hydroxyacids (Yuzawa et al., 2017). Short-chain 3-hydroxyacids are versatile compounds with many applications. Their unique chemical properties enable their use as monomers for producing polyhydroxyalkanoates, such as poly (3-hydroxybutyrate), which are used in biodegradable plastics, molded goods, adhesives, films, and coatings (Bannister and Prather, 2023). The chemical stability of hydroxy acids makes them suitable for use in high-performance lubricants and corrosion inhibitors for military and industrial equipment (Heng et al., 2024), and their amphiphilic properties make them useful in the formulation of coatings and surfactants (Cao and Zhang, 2013). Hydroxyacids are included in cosmetics as moisturizers, bactericides, and anti-inflammatory agents (Löwe and Gröger, 2020). Furthermore, medium-chain 3-hydroxyacids (e.g., 3-hydroxydecanoic acid) have been shown to induce systemic resistance in plants against pathogen infections, making them valuable for sustainable agriculture (Kutschera et al., 2019).

While there has been significant work to engineer microorganisms to produce 3-hydroxypropionic acid and other 3-hydroxyacids (K'yal and Prather, 2024; Song et al., 2016), the biosynthesis of each hydroxyacid requires a distinct biosynthesis pathway. Because of the versatility of T1PKSs, a single synthase can be engineered to produce a variety of hydroxyacids, either by exchanging the acyl transferase (AT) in the loading module and the extension module or by altering the acyl-CoAs supplied to the synthase (Yuzawa et al., 2017).

In this study, we successfully engineered our initial PKS design and expanded *P. putida*'s acyl-CoA pool for improved PKS-based 3-hydroxyacid production. We further showcase RB-TnSeq in the context of metabolic engineering and assess the impact of each modification under varying conditions.

## 2 Results

### 2.1 Phosphopantetheinyl transferase activity and polyketide extender supply

The basic requirement for the functional expression of a PKS is the activation of the acyl carrier protein (ACP) domain via phosphopantetheinylation. While it is known that *P. putida* possesses a broad-specificity PPTase, it was unclear whether the addition of a heterologous PPTase could improve T1PKS activity (Gross et al., 2005; Owen et al., 2011). To address this, we first integrated the PPTase gene *sfp* from *Bacillus subtilis* into the *P. putida* genome using a mini-Tn7 delivery transposon system (Zobel et al., 2015). All other heterologous genes were integrated into the genome using the serine-assisted genome engineering (SAGE) toolkit (Elmore et al., 2023).

Instead of measuring polyketide production directly, we opted to use the colorimetric blue-pigment synthase A (BpsA) assay to determine *in vivo* PPTase activity (Owen et al., 2011). The non-ribosomal peptide synthase (NRPS) BpsA converts two molecules of L-glutamine into the blue pigment indigoidine (Takahashi et al., 2007). The activation of its peptide carrier protein (PCP) domain by a PPTase is the rate-limiting step of indigoidine synthesis and serves as an indirect measure of PPTase activity (Owen et al., 2011).

**Fig. 1:**
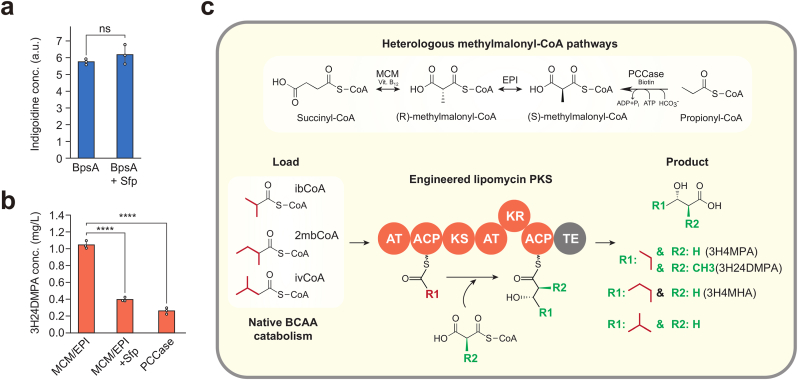
Engineered lipomycin polyketide synthase (LipPKS) expression in *Pseudomonas putida*. (a) The blue-pigment synthase A (BpsA) assay (blue) in *P. putida* with and without the addition of *sfp*. (b) 3-Hydroxy-2,4-dimethylpentanoic acid (3H24DMPA) production in engineered strains (orange). (c) General design of the engineered LipPKS pathway and its required precursors. The tested methylmalonyl-CoA (mmCoA) pathways comprised of either the mmCoA mutase and epimerase (MCM/EPI) from *Sorangium cellulosum* or the propionyl-CoA carboxylase (PCCase) complex from *Streptomyces coelicolor*. The preferred loading substrates are located in *P. putida*'s native branched-chain amino acid (BCAA) catabolism. The polyketide product is an enantiopure 3-hydroxyacid, which varies based on the substrate loaded. AT: acyl transferase; ACP: acyl carrier protein; KS: keto synthase; KR: keto reductase; TE: thioesterase; ibCoA: isobutyryl-CoA; 2mbCoA: 2-methylbutyryl-CoA; ivCoA: isovaleryl-CoA; 3H4MPA: 3-hydroxy-4-methylpentanoic acid; 3H4MHA: 3-hydroxy-4-methylhexanoic acid; (∗∗∗∗) = p < 0.0001; NS: non-significant. Error bars represent the standard deviation of n = 3.

Wild-type (WT) *P. putida* and *P. putida* Tn7Sfp expressing the *bpsA* gene produced a significant amount of indigoidine (Fig. 1a). The endogenous PPTase activity resulted in a normalized indigoidine signal of 5.8 ± 0.2 a. u. The strain with the additional PPTase Sfp showed no significant increase in indigoidine levels. Thus, by integrating a copy of *sfp* into the host genome, we were not able to improve indigoidine production.

To determine the effects of this modification on a T1PKS, we used an engineered version of the LipPKS that has been previously tested in a methylmalonyl-CoA-producing strain of *P. putida* (Schmidt et al., 2023). Production of the expected polyketide 3-hydroxy-2,4-dimethylpentanoic acid (3H24DMPA) could be observed in both strains (Fig. 1b). However, polyketide production levels in the *sfp*-expressing strain were 40 % of those in the WT strain. The endogenous PPTase activity in *P. putida* seems sufficient to activate the ACP domain of the expressed T1PKS protein. Therefore, we chose not to include an additional PPTase in our strains.

Subsequently, we focused on the availability of the common PKS extender unit, methylmalonyl-CoA (mmCoA). The genes encoding mmCoA mutase (MCM) and epimerase (EPI) from *Sorangium cellulosum* have been successfully expressed in *P. putida*, converting succinyl-CoA to mmCoA (Fig. 1c) (Gross et al., 2006b; Schmidt et al., 2023). However, the functional expression of the alternative propionyl-CoA carboxylase (PCCase) pathway, which comprises the *accA2* and *pccBE* genes from *Streptomyces coelicolor* and converts propionyl-CoA to mmCoA, has not been reported in this organism (Pfeifer et al., 2001).

In this study, we cloned and integrated those genes by using backbone excision-dependent expression (BEDEX) vectors (Schmidt et al., 2023). By including the repressor *lacI* into the backbone of the integrative vector and placing the target genes under the control of a *lac* promoter, toxicity or other burdensome effects can be mitigated. Once the backbone (with *lacI*) is excised, the *lac* promoter becomes constitutively active (Schmidt et al., 2023). In addition to utilizing BEDEX vectors, we also codon-optimized the *accA2* and *pccBE* genes using the “Harmonize Relative Codon Adaptiveness” (hrca) method (Schmidt et al., 2023; Zulkower and Rosser, 2020).

As a result, we confirmed the functional expression of this PCCase in *P. putida* by detecting the mmCoA-extended polyketide (Fig. 1b). Notably, the strain harboring the MCM/EPI pathway produced approximately 60 % more 3H24DMPA than the strain with the PCCase pathway.

*P. putida* has demonstrated its suitability as a host for the general assessment of engineered T1PKSs. The presence of a native, highly promiscuous PPTase, combined with the functional expression of either of the mmCoA pathways, enables the production of a wide range of polyketides.

### 2.2 BarSeq analysis of LipPKS starter unit metabolism

Target polyketide titers were low, which could be attributed to the availability of precursor acyl-CoAs. As the LipPKS loading pathway accepts branched acyl-CoAs that are intermediates in *P. putida*'s native BCAA metabolism (Fig. 1c), we decided to utilize RB-TnSeq to guide our engineering efforts to improve production of the preferred LipPKS starter units isobutyryl-CoA (ibCoA) and 2-methylbutyryl-CoA (2mbCoA) (Wetmore et al., 2015; Yuzawa et al., 2013).

**Fig. 2:**
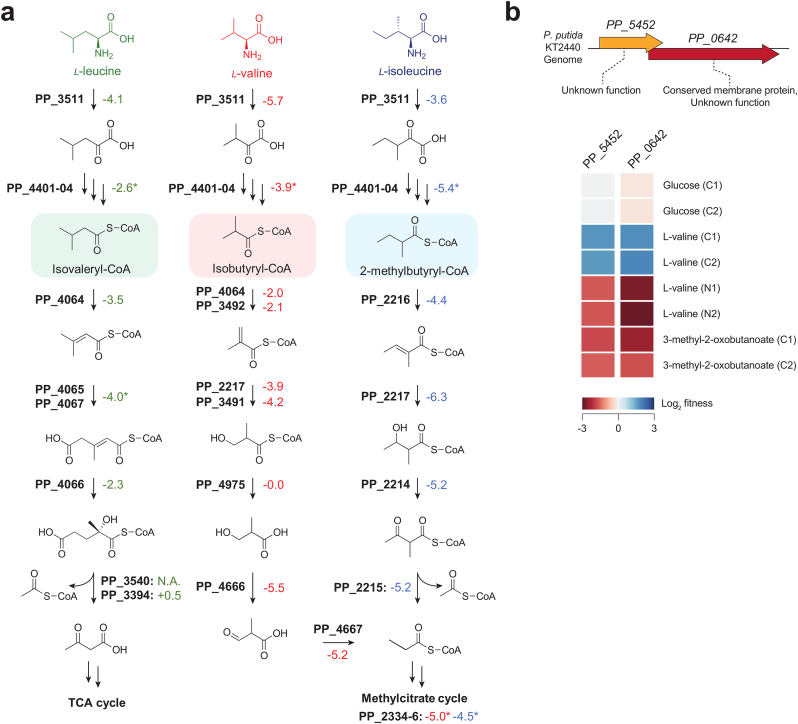
The branched-chain amino acid (BCAA) metabolism in *Pseudomonas putida*. (a) BarSeq analysis of the BCAA degradation pathway in *P. putida*. Fitness defects were considered significant when the fitness value was > |1| and the t-value was >5. Shown are the fitness values (n = 2) associated with growth on L-leucine (green), L-valine (red) and L-isoleucine (blue) as the sole source of carbon. Fitness values marked with an asterisk (∗) represent averages across multiple genes. (b) Genomic arrangement of PP_5452 and PP_0642 operon with heatmap of the associated fitness values (n = 2) for L-valine carbon source (C), 3-methyl-2-oxobutanoic acid (C) and L-valine nitrogen source (N) conditions.

To identify the genes responsible for the degradation of the LipPKS precursor acyl-CoAs, we fed L-leucine, L-valine, or L-isoleucine as the sole carbon source to a *P. putida* RB-TnSeq mutant library (Fig. 2a). To ensure that we identified all possible degradation pathways we included the corresponding 2-oxoacids for these BCAAs as well. The full dataset can be accessed through the public version of the Fitness Browser (https://fit.genomics.lbl.gov) (Price et al., 2018).

The resulting differences in mutant abundances were measured and used to calculate the fitness values and t-scores for conditionally essential genes (Wetmore et al., 2015). While the BCAA metabolism has been characterized previously by using them as a nitrogen source, their use as a carbon source has not been reported yet (Schmidt et al., 2022). The addition of BarSeq carbon source experiments can increase the significance for fitness data downstream from the deamination reaction and facilitates the identification of phenotypes for specific transporters (Schmidt et al., 2022).

The strongest transporter phenotypes we identified were for the genes PP_0878-PP_0881 and PP_1137-PP_1141, especially in the L-isoleucine and L-leucine conditions. While we could not identify a specific L-valine transporter, using L-valine as a carbon or nitrogen source and 3-methyl-2-oxobutanoic acid as a carbon source resulted in different growth phenotypes for the conserved proteins of unknown function PP_0642 and PP_5452 (Fig. 2b). Using L-valine as a carbon source, PP_0642 and PP_5452 have positive fitness values of 1.9 and 1.7, respectively. In contrast, using L-valine as a nitrogen source and 3-methyl-2-oxobutanoic acid as a carbon source resulted in similarly strong but negative fitness values. Based on these results, we propose that the gene product of PP_0642 (Q88Q56) is a transporter for 3-methyl-2-oxobutanoic acid. In addition, *in silico* structure predictions of Q88Q56 found on Uniprot show the potential existence of transmembrane domains. Although *P. putida* can utilize L-valine as the sole source of carbon and nitrogen, it appears to prefer L-valine as a nitrogen source. If an alternative source of carbon is available, the presence of PP_0642 could lead to the export of the corresponding 2-oxoacid rather than conversion to propionyl-CoA and carbon utilization by the methylcitrate cycle (Fig. 2b) (Hutchison et al., 2019; Rex et al., 1994).

Degradation of BCAAs seems to be initiated by the aminotransferase IlvE, which had a significant fitness defect of −4.5 across all three tested substrates. The subsequent step, which is catalyzed by the branched-chain α-keto acid dehydrogenase (BKD) complex (PP_4401-PP_4404), is shared across all the BCAA and 2-oxoacid conditions. This reaction also results in the targeted acyl-CoAs, ibCoA and 2mbCoA. Therefore, preventing the reaction downstream of the BKD complex should accumulate the desired LipPKS starter units.

Using BarSeq, we identified three acyl-CoA dehydrogenases (ACDHs), namely PP_4064, PP_3492 and PP_2216, potentially responsible for the degradation of ibCoA and 2mbCoA. The most essential ACDHs for ibCoA degradation appear to be PP_4064 (−2.0) and PP_3492 (−2.1), while 2mbCoA catabolism only requires PP_2216 (−4.4). To verify these results, we created a combinatorial library of deletion mutants for PP_4064, PP_3492 and PP_2216 and conducted growth experiments with BCAAs as the sole source of carbon (Supplementary Fig. 1). The identified ACDHs for isovaleryl-CoA (PP_4064) and 2mbCoA (PP_2216) were essential for growth on L-leucine and L-isoleucine, respectively. Furthermore, deletion of PP_3492 or PP_4064 did cause a significant growth defect when L-valine was used as the sole carbon source. However, a combination of both deletions improved growth slightly when compared to the single deletion of PP_3492. Finally, the triple deletion of PP_2216, PP_3492, and PP_4064 completely abolished growth on any of the three BCAAs. Therefore, strain ΔPP_2216ΔPP_3492ΔPP_4064 is potentially our best-performing host for branched chain polyketide production, as it cannot efficiently utilize any of the BCAAs as growth substrates.

While we successfully identified all the relevant ACDHs involved in the metabolism of BCAAs, a well-known limitation of BarSeq experiments is their inability to identify phenotypes when there are redundant genes for a particular enzymatic activity (Wetmore et al., 2015). If redundant gene function is expected, it is important to verify BarSeq results by classical mutant growth experiments (Supplementary Fig. 1).

### 2.3 Production of des-methyl 3-hydroxyacids

One of the key advantages of producing chemicals using T1PKSs is the ability to make a variety of similar molecules by varying the domains in the PKS or by altering the pools of precursors made by the cells. As subtle changes in the resulting product can affect its properties, PKSs can be modified to tailor chemical properties. For 3-hydroxyacids, the initially tested version of the LipPKS contains a mmCoA-specific AT domain, which adds a methyl group to the α-carbon position of the polyketide (Fig. 1c). As mmCoA is not naturally present in *P. putida* and could become a limiting factor, the PKS extender malonyl-CoA (mCoA) presents a more suitable alternative for achieving higher polyketide titers. Consequently, we exchanged the native AT of the first extension module of LipPKS for an mCoA-accepting AT. Based on previous results, we chose the highly promiscuous ansamycin PKS module 8 (AnsM8) AT and the mCoA-specific AT from the borrelidin PKS module 1 (BorM1) (Englund et al., 2023b). Additionally, we included the native AT due to its recently reported *in vivo* activity with mCoA (Schmidt et al., 2023). AT-exchanges were performed using the updated domain boundaries (us3/ds44) identified by Englund et al. (2023a)

The best-performing AT-exchanged mutant contained the AnsM8-AT, resulting in 60 % higher titers of 3-hydroxy-4-methylpentanoic acid (3H4MPA) compared to the native AT (Supplementary Fig. 2). Over a 120-h period, the titers of 3H4MPA were relatively constant with a very slight increase towards the end of the cultivation. Interestingly, the PKS engineered with the BorM1 AT did not produce any detectable product. This might be due to an incompatible exchange junction. Previously, we showed that the LipPKS containing the BorM1 AT produced a 3-hydroxy acid (Yuzawa et al., 2017). However, that functional AT-exchanged PKS used the us1/ds44 junction rather than the us3/ds44 junction that we used here. Junction boundaries are most likely not universally applicable to every PKS acceptor and donor pair and might require individual assessment.

### 2.4 Altering malonyl-CoA levels using protein degradation tags

Due to its role in central metabolism, concentration of the common polyketide precursor mCoA is a challenging target to manipulate. Current strategies mainly focus on downregulating the expression of enzymes that consume it or overexpressing enzymes that produce it, namely the carboxylation reaction of acetyl-CoA (Milke and Marienhagen, 2020). An underutilized strategy is the post-translational control of protein levels. By exploiting widely distributed and native protease complexes such as ClpXP, essential proteins can be artificially tagged with a short peptide sequence (>20 aa) for degradation and thereby reduce their steady-state levels inside the cell (Li et al., 2023). To avoid rapid and almost instantaneous degradation by ClpXP, these so called ssrA tags require minimal modifications to either make ClpXP recognition SspB-dependent or decrease degradation rates (McGinness et al., 2006). Furthermore, compared to conventional methods, ssrA-tagging does not require heterologous gene expression and has an immediate effect on protein levels.

**Fig. 3:**
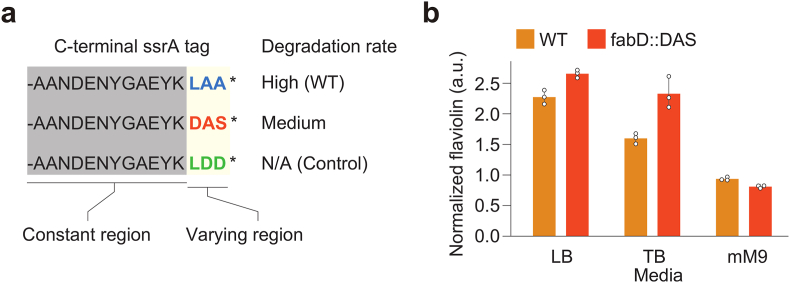
Protein degradation tags as a tool to control malonyl-CoA (mCoA) levels in *Pseudomonas putida*. (a) Engineered ssrA tags and their corresponding degradation rates in *P. putida*. (b) Flaviolin production by the mCoA reporter RppA in modified *P. putida* strains with ssrA-tagged malonyl-CoA:ACP transacylase (FabD). WT: Wild-type; LB: Luria-Bertani medium; TB: Terrific Broth; mM9: modified M9 medium. Error bars represent the standard deviation of n = 3.

To better understand this system in *P. putida*, we identified its native ssrA tag sequence (GenBank: CDI33222.1) and replaced the ClpXP recognition site, Leu-Ala-Ala (LAA), with Asp-Ala-Ser (DAS) and Leu-Asp-Asp (LDD) (Fig. 3a). These modified ssrA tags were first tested on heterologously expressed green fluorescent protein (GFP) for degradation (Andersen et al., 1998) (Supplementary Fig. 3). As expected, the WT ssrA-variant, LAA, resulted in lower levels of GFP accumulating inside the cell (e.g., rapid degradation), the LDD tag had no influence on GFP levels (the same intracellular level as the untagged GFP), and the DAS tag resulted in an intermediate level of GFP inside the cell (indicating a degradation rate between the LAA tag and the LDD tag). Indeed, it has been shown that replacing the hydrophobic Ala residues with electrically charged amino acids completely abolishes ClpXP recognition (McGinness et al., 2006), so the lack of change in GFP levels for protein tagged with LDD is not surprising. In contrast to the reported SspB-dependency in *E. coli*, the engineered DAS tag seems to lead to a constitutive, medium rate of degradation in *P. putida* (McGinness et al., 2006).

To demonstrate the usefulness of the newly identified ssrA tag, we C-terminally attached the DAS tag to the malonyl-CoA:ACP transacylase FabD (PP_1913), which catalyzes the essential first step of fatty acid biosynthesis. While no differences in polyketide titers could be observed (data not shown), the mCoA reporter RppA showed a significant increase in flaviolin production when FabD was tagged for protease degradation (p < 0.05) (Incha et al., 2020). Interestingly, this increase could only be observed in Luria-Bertani (LB) or Terrific Broth (TB) medium (Fig. 3b). It is likely that in the case of polyketide production by our type I system, titers are not limited by mCoA availability. While decreasing protein levels of FabD did not lead to improved titers of the target polyketide, we showed that it has a significant effect on the highly mCoA-dependent production of flaviolin (Yang et al., 2018). SsrA-tagging could be a powerful tool to downregulate the presence of essential gene products and avoid the unnecessary burden of heterologous systems.

### 2.5 Media optimization and strain assessment

**Fig. 4:**
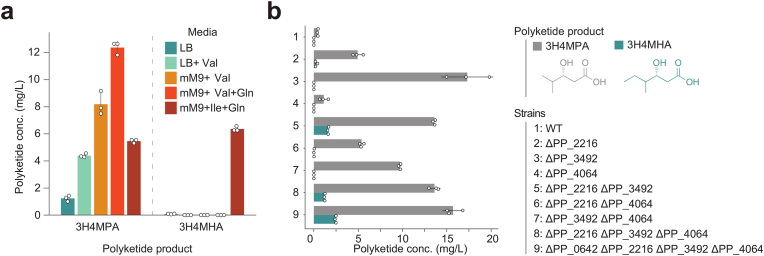
Optimization of polyketide titers in *Pseudomonas putida*. (a) Media optimization for the polyketides 3-hydroxy-4-methylpentanoic acid (3H4MPA) (left) and 3-hydroxy-4-methylhexanoic acid (3H4MHA) (right). (b) Polyketide production in combinatorial library of strains deficient for acyl-CoA dehydrogenases (ACDHs) PP_2216, PP_3492, or PP_4064. In addition, a deletion of the potential 3-methyl-2-oxobutanoic acid transporter (PP_0642) was tested in strain ΔPP_2216ΔPP_3492ΔPP_4064. LB: Luria-Bertani medium; mM9: modified M9 medium; WT: wild-type. Error bars represent the standard deviation of n = 3.

The next step focused on media optimization (Fig. 4a). Our standard screening medium for LipPKS polyketide production in *P. putida* is LB supplemented with 20 mM L-valine. The addition of L-valine most likely results in higher nitrogen content and better ibCoA availability. Contrary to our expectations, changing our standard screening medium from a rich medium (LB) to a modified version of the minimal medium M9 (mM9), which contains FeSO_4_ and 100 mM glucose, led to significantly improved polyketide titers (+46 %) (Johnson et al., 2016). The additional FeSO_4_ reduces iron-starvation, which is a known stress factor in fluorescent pseudomonads, and high glucose concentrations have been shown to increase mCoA availability (Incha et al., 2020; Sasnow et al., 2016). To further improve production, we added 10 mM L-glutamine as an additional source of nitrogen. L-Glutamine has shown to have superior characteristics as a nitrogen source for *P. putida*, and nitrogen starvation seems to be a common issue for this bacterium (Schmidt et al., 2022). When compared to LB + Val, the final medium, designated as mM9+Val + Gln, increased 3H4MPA titers three-fold. Furthermore, by replacing L-valine with L-isoleucine, we were able to substantially boost 3-hydroxy-4-methylhexanoic acid (3H4MHA) titers from undetectable in LB + Val to 6.4 ± 0.2 mg/L in mM9+Ile + Gln. To our surprise, the same medium also led to the production of the isobutyryl-based polyketide 3H4MPA (5.5 ± 0.1 mg/L) without the supplementation of L-valine.

The next step focused on the effects of the ACDH deletions and, therefore, the availability of the acyl-CoA loading substrate. Polyketide production in the combinatorial ACDH-deficient strains and mM9+Val + Gln medium is shown in Fig. 4b. Although no L-isoleucine was supplemented to the medium, the strain that had the gene encoding the ACDH PP_2216 deleted (strain ΔPP_2216) successfully produced the 2-methylbutyryl-based polyketide, 3H4MHA. This result is consistent with the growth of ΔPP_2216 on L-isoleucine as the sole carbon source (Supplementary Fig. 1). Surprisingly, the additional deletion of PP_3492 (strain ΔPP_2216ΔPP_3492) resulted in a 6-fold increase in 3H4MHA titers. The final strain ΔPP_0642ΔPP_2216ΔPP_3492ΔPP_4064 achieved the highest titer of 2.5 ± 0.1 mg/L 3H4MHA.

As expected in a L-valine supplemented medium, the titers for the isobutyryl-based polyketide, 3H4MPA, were significantly higher than 3H4MHA titers. With a combined polyketide titer of 18.2 ± 0.1 mg/L, strain ΔPP_0642ΔPP_2216ΔPP_3492ΔPP_4064 performed slightly better than the second-best strain, ΔPP_3492. The additional deletion of the newly identified transporter PP_0642 in the ΔPP_2216ΔPP_3492ΔPP_4064 strain led to improved titers for 3H4MPA and 3H4MHA, respectively. Although RB-TnSeq and growth data with L-valine as the sole source of carbon indicated that PP_4064 deletion should impact polyketide titers, its deletion did not have any significant impact on them. While growth might be affected by the deletion of PP_4064, it might not affect intracellular ibCoA concentrations.

Overall, the deletion of the RB-TnSeq identified target genes resulted in significant titer improvements and also enabled the *de novo* production of the 2-methylbutyryl-based polyketide 3H4MHA.

## 3 Discussion

In this study we established *P. putida* as a non-native host for T1PKSs. Through rational metabolic engineering, we were able to improve the supply of common polyketide precursors and increase product titers.

While Sfp is a well-established heterologous PPTase for PKS expression, we were able to demonstrate that it is not necessary in *P. putida* and can even result in decreased polyketide titers (Pfeifer et al., 2001). Previously, it was shown that adding an additional PPTase to a non-native PKS host, leads to the depletion of Mg^2+^-ions and negatively affects growth (Kallscheuer et al., 2019).

Another aspect of establishing *P. putida* as a host for T1PKSs is the supply of the very common PKS extender unit, mmCoA. Due to the lack of this pathway in *P. putida*, Gross et al. enabled the biosynthesis of mmCoA using the MCM/EPI pathway, which utilizes succinyl-CoA as the precursor. In this study, we were also able to express the commonly used PCCase pathway. In *E. coli*, expression of the PCCase from *S. coelicolor* resulted in the highest mmCoA-dependent polyketide titers (Vandova et al., 2017). Besides integrating *accA2* and *pccB,* we included the lesser known ε-subunit, PccE, which has been shown to increase *in vitro* activity of the PCCase complex (Diacovich et al., 2002). Although our final polyketide titers in *P. putida* expressing the PCCase pathway were lower than in the MCM/EPI pathway containing strain, the PCCase pathway is generally recognized as superior (Vandova et al., 2017). The supply of propionyl-CoA or slower growth of cells expressing the PCCase might be potential bottlenecks. Finally, a combination of both mmCoA pathways could be a novel approach to further increase intracellular mmCoA levels in *P. putida*.

With our initial evaluation of *P. putida* as a host for T1PKSs, we could successfully confirm that an additional PPTase is not needed. Furthermore, we ensured the supply of the common PKS extender unit mmCoA and enabled the functional expression of the PCCase pathway as an alternative to currently available pathways in this host.

One of the advantages of using *P. putida* as a host for production of chemicals is its ability to catabolize a variety of carbon sources; however, it is also able to consume many products that we want to produce or the metabolic intermediates to those products, such as ibCoA and 2mbCoA. While these branched acyl-CoAs are natively present in *P. putida*'s BCAA catabolism, we determined that their supply is a key factor for improving *in vivo* polyketide titers. To perform precise modifications to the metabolism of *P. putida*, we analyzed its BCAA degradation pathway using RB-TnSeq (Wetmore et al., 2015). In the context of rational metabolic engineering, BarSeq has already proven to be an invaluable tool for deciphering complex metabolisms such as that of *P. putida* (Thompson et al., 2019b). In our study, BarSeq facilitated the identification of essential genes that are involved in BCAA metabolism. Besides confirming known gene functions, we were also able to identify the previously unknown ibCoA dehydrogenase PP_3492, which is one of our three target ACDHs. In addition, we might have discovered the function of the hypothetical protein for PP_0642. Based on our findings related to the specific growth phenotypes for the L-valine and 3-methyl-2-oxobutanoic acid condition, we propose that PP_0642 (Q88Q56) is a transporter involved in the export and uptake of 3-methyl-2-oxobutanoic acid. According to the Uniprot database, this finding is also supported by the predicted structure of Q88Q56.

A possible explanation for the positive growth phenotype in the L-valine condition, could be the active export of the deaminated C4 intermediate by PP_0642. If 3-methyl-2-oxobutanoic acid is not exported, the subsequent downstream reactions result in the uneven fatty acyl-CoA, propionyl-CoA. The buildup of propionyl-CoA metabolites can have a negative effect on growth and it might be beneficial for the bacterium to utilize exogenous L-valine strictly as a nitrogen source (Upton and McKinney, 2007).

The final verification of the RB-TnSeq data via mutant growth assays demonstrated the successful identification of the ACDHs downstream from our target acyl-CoAs. The deletion of PP_2216, PP_3492 and PP_4064 completely abolished growth on any supplemented BCAA. To show that we accumulate the corresponding acyl-CoAs, we tested an engineered version of the LipPKS in a combinatorial ACDH mutant library. As a result, we identified the strain ΔPP_0642ΔPP_2216ΔPP_3492ΔPP_4064 as our top-performer. The use of RB-TnSeq in metabolic engineering is a powerful tool to guide the optimization of a host metabolism and can help to facilitate the host engineering process.

Throughout the optimization process, the medium composition seemed to have the greatest impact on polyketide titers. To our surprise, the minimal medium M9 supplemented with amino acids and glucose led to significantly higher polyketide titers than the rich medium, LB. We hypothesize that the limited number of different carbon sources might increase the flux towards mCoA and facilitate carbon utilization of the deaminated amino acids. Subsequently, the higher availability of polyketide precursors leads to increased polyketide titers.

While tagging the essential malonyl-CoA:ACP transacylase FabD with an engineered protein degradation tag did not lead to improved polyketide titers, we successfully showed its impact on the production of flaviolin. Downregulating protein activity using engineered ssrA tags is a less common strategy in metabolic engineering, with only a few examples in *P. putida* (Batianis et al., 2023). The basic mechanism of protein degradation is present in a wide range of organisms and does not require the expression of heterologous genes. After the identification of suitable ssrA tags, it can be easily exploited to control the levels of essential proteins.

Regardless of the low titers, engineered PKSs can be used as highly flexible and versatile platforms for the production of specialty chemicals (Dan et al., 2025; Yuzawa et al., 2018). The branched short-chain fatty acids we produced could be easily further modified towards completely new characteristics, highly sought after in materials or pharmaceuticals.

## 4 Materials and methods

### 4.1 Chemicals, media and culture conditions

All chemicals used throughout this study were purchased from Sigma-Aldrich (USA) unless otherwise described. Authentic standards for 3H24DMPA, 3H4MPA, and 3H4MHA were synthesized by Enamine (Ukraine). Precultures of *E. coli* and *P. putida* were grown from single colonies in LB medium supplemented with 50 μg/mL kanamycin or 20 μg/mL chloramphenicol, respectively. *E. coli* cultures were cultured at 37 °C, while the temperature for *P. putida* cultures was set to 30 °C. The shaking speed was maintained at 200 rpm. Main cultures of *P. putida* were either grown in 30 mL glass tubes or 24-well plates (VWR, USA), and inoculated with a 1:100 (v/v) ratio of medium to overnight culture. Prior to inoculation, overnight cultures were washed twice with the final medium. Other media used in this study were TB, MOPS and mM9 (Johnson et al., 2016; LaBauve and Wargo, 2012). The mM9 medium consists of 13.56 g/L Na_2_HPO_4_, 6 g/L KH_2_PO_4_, 1 g/L NaCl, 2 g/L NH_4_Cl, 2 mM MgSO_4_, 100 μM CaCl_2_, and 18 μM FeSO_4_, and 20–100 mM glucose. If applicable, the mM9 medium was supplemented with 10 mM L-glutamine and 20 mM L-valine or L-isoleucine, respectively. To prevent excessive iron oxidation, 1000× FeSO_4_ stock solutions were prepared fresh and added right before inoculation.

### 4.2 Plasmid and strain construction

Plasmids and strains used in this study are listed in Supplementary Tables 1 and 2 All strains and plasmids generated in this work are publicly available through the JBEI registry (https://public-registry.jbei.org/folders/887). Plasmids and primers for construction were designed using the j5 DNA assembly automation software (Hillson et al., 2012). Primers and synthetic DNA sequences were purchased from Integrated DNA Technologies (USA) or Genscript (USA). Prior to DNA synthesis, DNA sequences were codon optimized using BaseBuddy (https://basebuddy.lbl.gov) (Schmidt et al., 2023). For T4 DNA ligations (NEB, USA), vector backbones and inserts were digested using *Nde*I and *Xho*I or *Bam*HI, respectively (NEB, USA). Plasmid extractions were performed following the instructions of the Qiaprep Spin Miniprep kit (Qiagen, Germany).

All genetically modified strains of *P. putida* were based on the polyAttB strain AG5577 (Schmidt et al., 2023)*.* Strains with in-frame gene deletions were generated via homologous recombination as previously described (Thompson et al., 2019a). Gene integrations were either achieved by Tn7 delivery transposons or serine recombinases (Elmore et al., 2023; Zobel et al., 2015). Successful gene deletions or integrations were confirmed using colony PCR (cPCR).

### 4.3 Plate-based growth assays

Bacterial growth studies were carried out using plate reader kinetic assays, following the methodology described in Thompson et al. (2019a). First, overnight cultures of *P. putida* strains were washed three times with carbon-free mM9 minimal medium. These cultures were then used to inoculate 48-well plates (VWR, USA) at a ratio of 1:100, with each well containing 500 μL of mM9 medium supplemented with 10 mM of the tested carbon source. The OD of the cultures was monitored for up to 72 h at 30 °C using a Biotek Synergy H1M plate reader (BioTek, USA) set to fast continuous shaking. The OD was measured at 600 nm. Optionally, GFP levels were measured with an excitation wavelength of 395 nm and an emission wavelength of 509 nm.

### 4.4 Phosphopantetheinyl transferase and malonyl-CoA reporter assay

Strains containing the gene for BpsA were grown for 24 h in LB, and harvested by centrifugation. The pellet was then resuspended in an equal volume of DMSO. Next, the mixture was vortexed for 10 min at 3000 rpm, followed by centrifugation at maximum speed for 1 min 100 μL of the supernatant was then transferred into a black, clear-bottom 96-well plate. If necessary, samples were diluted with water. The indigoidine concentration was estimated by measuring the absorbance of the supernatant at 612 nm with a Biotek Synergy H1M plate reader (BioTek, USA) (Wehrs et al., 2018).

Strains carrying the pBADT-RppA vector were cultured for 24 h in LB, TB and mM9, respectively, containing 0.2 % L-arabinose and 50 μg/mL kanamycin. Samples were harvested by a 1-min centrifugation at maximum speed. 100 μL of the supernatant was then transferred into a black, clear-bottom 96-well plate. The flaviolin concentration was estimated by measuring the absorbance of the supernatant at 340 nm with a Biotek Synergy H1M plate reader (BioTek, USA) (Incha et al., 2020).

All strains were cultivated in triplicates and final indigoidine and flaviolin absorbance was normalized by OD600.

### 4.5 BarSeq assays

BarSeq experiments used the *P. putida* library JBEI-1 derived from Rand et al., following methods previously described in Thompson et al. (Rand et al., 2017; Thompson et al., 2019a). Briefly, a 2 mL aliquot of the JBEI-1 library was thawed on ice, mixed into 25 mL of LB medium with 50 μg/mL kanamycin, and cultivated at 30 °C until an OD600 of 0.5. Subsequently, three 1-mL aliquots were removed, pelleted, and stored at −80 °C as initial time points. Next, the library culture was triple washed with carbon-free MOPS minimal medium and used to inoculate each media at a 1:100 ratio. Selective growth was carried out in 24-well plates with 2 mL of MOPS minimal medium containing 10 mM of the tested carbon source. Plates were cultivated at 30 °C with a shaking speed of 200 rpm. After 24–72 h, 1 mL samples were harvested when the cultures seemed dense enough for DNA extraction. These samples were pelleted and kept at −80 °C until DNA was extracted using the DNeasy UltraClean Microbial kit (Qiagen, Germany). BarSeq analysis was conducted as outlined in Wetmore et al. (2015) Statistic t-values > |5| and fitness values > |1| were regarded as significant. BarSeq experiments were carried out as duplicates and fitness data is publicly available at http://fit.genomics.lbl.gov.

### 4.6 Polyketide production and LC-MS analysis

After 48 h of growth, samples were harvested and quenched with an equal volume of −80 °C methanol. This mixture was then centrifuged at maximum speed and the supernatant was filtered using 3 kDa Amicon Ultra Centrifugal Filters (Millipore, USA) at 4 °C. Next, the flow-through was collected in GC-vials (Agilent, USA) and stored at −80 °C until further analysis. A detailed LC-MS method has been described previously (Schmidt et al., 2023).

## CRediT authorship contribution statement

**Matthias Schmidt:** Writing – review & editing, Writing – original draft, Visualization, Methodology, Investigation, Conceptualization. **Aaron A. Vilchez:** Writing – review & editing, Investigation. **Namil Lee:** Writing – review & editing, Visualization, Investigation. **Leah S. Keiser:** Writing – review & editing, Investigation. **Allison N. Pearson:** Writing – review & editing, Investigation. **Mitchell G. Thompson:** Writing – review & editing, Investigation. **Yolanda Zhu:** Writing – review & editing, Investigation. **Robert W. Haushalter:** Writing – review & editing, Investigation. **Adam M. Deutschbauer:** Writing – review & editing, Investigation. **Satoshi Yuzawa:** Writing – review & editing, Investigation. **Lars M. Blank:** Writing – review & editing, Supervision. **Jay D. Keasling:** Writing – review & editing, Supervision.

## Declaration of competing interest

The authors declare the following financial interests/personal relationships which may be considered as potential competing interests: Jay D. Keasling has financial interests in Ansa Biotechnologies, Apertor Pharma, Berkeley Yeast, Demetrix, Lygos, Napigen, ResVita Bio, and Zero Acre Farms.

## Data Availability

Strains, plasmids and RB-TnSeq data are available via public websites. The URL for those websites is mentioned in the manuscript.
