# Supplementary material for: Engineering Pseudomonas putida for production of 3-hydroxyacids using hybrid type I polyketide synthases
Source: Metab Eng Commun. 2025 Apr 2;20:e00261. doi: 10.1016/j.mec.2025.e00261 (PMC12005932; doi:10.1016/j.mec.2025.e00261)
Supplement: Multimedia component 1 [file mmc1.pdf]

Supplementary Materials

Engineering *Pseudomonas putida* for production of 3-hydroxyacids using hybrid type I polyketide synthases

Matthias Schmidt[1,2,3,4], Aaron A. Vilchez[1,2,9], Namil Lee[1,2,4], Leah S. Keiser[1,2,9], Allison N. Pearson[1,2,5], Mitchell G. Thompson[1,8], Yolanda Zhu[1,2,9], Robert W. Haushalter[1,2], Adam M. Deutschbauer[5,8], Satoshi Yuzawa[1,2,10,11], Lars M. Blank[3], Jay D. Keasling[1,2,4,6,7,8,9,12*]

[1]Joint BioEnergy Institute, 5885 Hollis Street, Emeryville, CA 94608, USA.
[2]Biological Systems & Engineering Division, Lawrence Berkeley National Laboratory, Berkeley, CA 94720, USA.
[3]Institute of Applied Microbiology (iAMB), Aachen Biology and Biotechnology (ABBt), RWTH Aachen University, Aachen, Germany
[4]California Institute for Quantitative Biosciences (QB3), University of California, Berkeley, CA 94720, USA
[5]Department of Plant and Microbial Biology, University of California, Berkeley, CA 94720, USA
[6]Joint Program in Bioengineering, University of California, Berkeley/San Francisco, CA 94720, USA
[7]Department of Chemistry, University of California, Berkeley, CA 94720, USA
[8]Environmental Genomics and Systems Biology Division, Lawrence Berkeley National Laboratory, Berkeley, CA 94720, USA
[9]Department of Chemical and Biomolecular Engineering, University of California, Berkeley, CA 94720, USA
[10]Systems Biology Program, Graduate School of Media and Governance, Keio University, Fujisawa, Kanagawa 252-0882, Japan
[11]Institute of Advanced Biosciences, Keio University, Tsuruoka, Yamagata 997-0017, Japan
[12]The Novo Nordisk Foundation Center for Biosustainability, Technical University of Denmark, Denmark

*Corresponding author: Jay D. Keasling, jdkeasling@lbl.gov

Table 1: Plasmids used in this study.

Plasmid	Description	Reference	JBEI part ID
pMQ30k	Suicide vector for allelic replacement with KanR, SacB	(Shanks et al., 2006)	
pMQ30k ΔPP_2216	In-frame deletion of PP_2216.	This work.	JPUB_026083
pMQ30k ΔPP_3492	In-frame deletion of PP_3492.	This work.	JPUB_026085
pMQ30k ΔPP_4064	In-frame deletion of PP_4064.	This work.	JPUB_026087
pMQ30k ΔPP_0642	In-frame deletion of PP_0642.	This work.	JPUB_026089
pMQ30k fabD::DAS-tag	C-terminal attachment of the DAS-tag for ClpXP degradation.	This work.	JPUB_026091
pBG14e GFP	Tn7 integration vector with oriR6K, KanR, FRT sites, Tn7L and Tn7R extremes and synthetic promoter 14e driving GFP	(Zobel et al., 2015)	
pBG14e Sfp	Integration of Pp_mcu codon optimized sfp from Bacillus subtilits.	This work.	JPUB_026093
pBG14e GFP::DAS-tag	Integration of C-terminally ssrA-tagged GFP.	This work.	JPUB_026095
pBG14e GFP::LDD-tag	Integration of C-terminally ssrA-tagged GFP.	This work.	JPUB_026097

pBG14e GFP::LAA-tag	Integration of C-terminally ssrA-tagged GFP	This work.	JPUB_026099
pTnS-1	oriR6K vector with AmpR and tnSABC+D operon	(Choi et al., 2005)	
pRK2013	RK2-derived plasmid with ColE1, KanR and tra+mob+	(Figurski and Helinski, 1979)	
pBADT	Broad host-range vector with BBR1 origin, arabinose inducible promoter, and KanR	(Bi et al., 2013)	
pBADT RppA	Expression of mCoA reporter RppA.	(Incha et al., 2020)	
pBH026	BxB1 integration vector with promoter LlacO1 repressed by LacI. KanR, ColE1 origin and LacI are flanked by ΦC31 attB and attP site.	(Schmidt et al., 2023)	
pBH026 BpsA	Integration of bpsA gene from Streptomyces lavendulae.	This work.	JPUB_026101
pBH026 lipPks1-TE	Integration of Pp_mcu codon optimized lipPks1 fused to EryM6-TE.	(Schmidt et al., 2023)	
pBH026 lipPks1(BorM1-AT)-TE	Integration of lipPks1-TE with BorM1-AT-exchange. Sequence for BorM1-AT is Pp_mcu codon optimized.	This work.	JPUB_026103
pBH026 lipPks1(AnsM8-AT)-TE	Integration of lipPks1-TE with AnsM8-AT-exchange. Sequence for AnsM8-AT is Pp_mcu codon optimized.	This work.	JPUB_026105

pBH027	Derivative of pBH026 with RV attP site.	(Schmidt et al., 2023)	
pBH027 PCCase	Integration of the Pp_hrca codon optimized genes accA2 and pccBE from Streptomyces coelicolor	This work.	JPUB_026107
pJH209	MR11 integration vector containing MR11 attP site. Kanamycin selection marker and ColE1 origin are flanked by ΦC31 attB and attP site.	(Elmore et al., 2023)	
pJH209t Sc_mmCoA	Integration of mmCoA pathway from S. cellulosum So56 under the control of constitutive P_{tac}	(Schmidt et al., 2023)	
pGW30	ColE1 vector with apramycin marker expressing ΦC31 integrase under the control of constitutive P_{tac}	(Elmore et al., 2023)	
pGW31	Derivative of pGW30 with BxB1 integrase.	(Elmore et al., 2023)	
pGW32	Derivative of pGW30 with RV integrase.	(Elmore et al., 2023)	
pGW36	Derivative of pGW30 with MR11 integrase.	(Elmore et al., 2023)	

Table 2: Strains used in this study.

Strain	Description	Reference	JBEI part ID
E. coli XL1 Blue	General cloning work.	Agilent	
E. coli HB101	Helper strain for Tn7 integration into P. putida.	(Boyer and Roulland-Dussoix, 1969)	
E. coli PIR2	Carrying Tn7 integration vector.	Thermo Fisher Scientific	
E. coli DH5α-λpir	Helper strain for Tn7 integration into P. putida.	Thermo Fisher Scientific	
P. putida AG5577	Derivative of P. putida KT2440 with three triple attB sites replacing PP_4740 and PP_2876 and integrated between PP_4217/PP_4218, respectively.	Adam Guss, personal communication.	
	Tn7 integrated pBG14e GFP	This work.	JPUB_026108
	Tn7 integrated pBG14e GFP::DAS-tag	This work.	JPUB_026110
	Tn7 integrated pBG14e GFP::LDD-tag	This work.	JPUB_026111
	Tn7 integrated pBG14e GFP::LAA-tag	This work.	JPUB_026112
	Carrying pBADT RppA	This work.	JPUB_026113
	BxB1 integrated pBH026 BpsA. pBH026 backbone excised.	This work.	JPUB_026114
	BxB1 integrated pBH026 BpsA and Tn7 integrated pBG14 Sfp.	This work.	JPUB_026115

	Only pBH026 backbone excised.		
	BxB1 integrated pBH026 lipPks1(BorM1-AT)-TE. pBH026 backbone excised.	This work.	JPUB_026116
	BxB1 integrated pBH026 lipPks1(AnsM8-AT)-TE. pBH026 backbone excised.	This work.	JPUB_026117
	BxB1 integrated pBH026 lipPks1-TE. pBH026 backbone excised.	(Schmidt et al., 2023)	
	BxB1 integrated pBH026 lipPks1-TE and RV integrated pBH027 PCCase. Both vector backbones excised.	This work.	JPUB_026118
P. putida AG5577mm	MR11 integrated pJH209 Sc_mmCoA. pJH209 backbone excised.	(Schmidt et al., 2023)	
	BxB1 integrated pBH026 lipPks1-TE. pBH026 backbone excised.	(Schmidt et al., 2023)	
	BxB1 integrated pBH026 lipPks1-TE and Tn7 integrated pBG14e Sfp. Only pBH026 backbone excised.	This work.	JPUB_026119
P. putida AG5577 fabD::DAS-tag	Derivative of AG5577 with ssrA-tagged FabD for ClpXP degradation	This work.	JPUB_026136
	Carrying pBADT RppA	This work.	JPUB_026137

	BxB1 integrated pBH026 lipPks1(AnsM8-AT)-TE. pBH026 backbone excised.	This work.	JPUB_026138
P. putida AG5577 ΔPP_2216	Derivative of AG5577 with in-frame deletion of PP_2216	This work.	JPUB_026120
	BxB1 integrated pBH026 lipPks1(AnsM8-AT)-TE. pBH026 backbone excised.	This work.	JPUB_026121
P. putida AG5577 ΔPP_3492	Derivative of AG5577 with in-frame deletion of PP_3492	This work.	JPUB_026122
	BxB1 integrated pBH026 lipPks1(AnsM8-AT)-TE. pBH026 backbone excised.	This work.	JPUB_026123
P. putida AG5577 ΔPP_4064	Derivative of AG5577 with in-frame deletion of PP_4064	This work.	JPUB_026124
	BxB1 integrated pBH026 lipPks1(AnsM8-AT)-TE. pBH026 backbone excised.	This work.	JPUB_026125
P. putida AG5577 ΔPP_2216ΔPP_3492	Derivative of AG5577 with in-frame deletions of PP_2216 and PP_3492	This work.	JPUB_026126
	BxB1 integrated pBH026 lipPks1(AnsM8-AT)-TE. pBH026 backbone excised.	This work.	JPUB_026127
P. putida AG5577 ΔPP_2216ΔPP_4064	Derivative of AG5577 with in-frame deletions of PP_2216 and PP_4064.	This work.	JPUB_026128

	BxB1 integrated pBH026 lipPks1(AnsM8-AT)-TE. pBH026 backbone excised.	This work.	JPUB_026129
P. putida AG5577 ΔPP_3492ΔPP_4064	Derivative of AG5577 with in-frame deletions of PP_3492 and PP_4064.	This work.	JPUB_026130
	BxB1 integrated pBH026 lipPks1(AnsM8-AT)-TE. pBH026 backbone excised.	This work.	JPUB_026131
P. putida AG5577 ΔPP_2216ΔPP_3492ΔPP_4064	Derivative of AG5577 with in-frame deletions of PP_2216, PP_3492, and PP_4064.	This work.	JPUB_026132
	BxB1 integrated pBH026 lipPks1(AnsM8-AT)-TE. pBH026 backbone excised.	This work.	JPUB_026133
P. putida AG5577 ΔPP_2216ΔPP_3492ΔPP_4064ΔPP_0642	Derivative of AG5577 with in-frame deletions of PP_2216, PP_3492, PP_4064, and PP_0642	This work.	JPUB_026134
	BxB1 integrated pBH026 lipPks1(AnsM8-AT)-TE. pBH026 backbone excised.	This work.	JPUB_026135

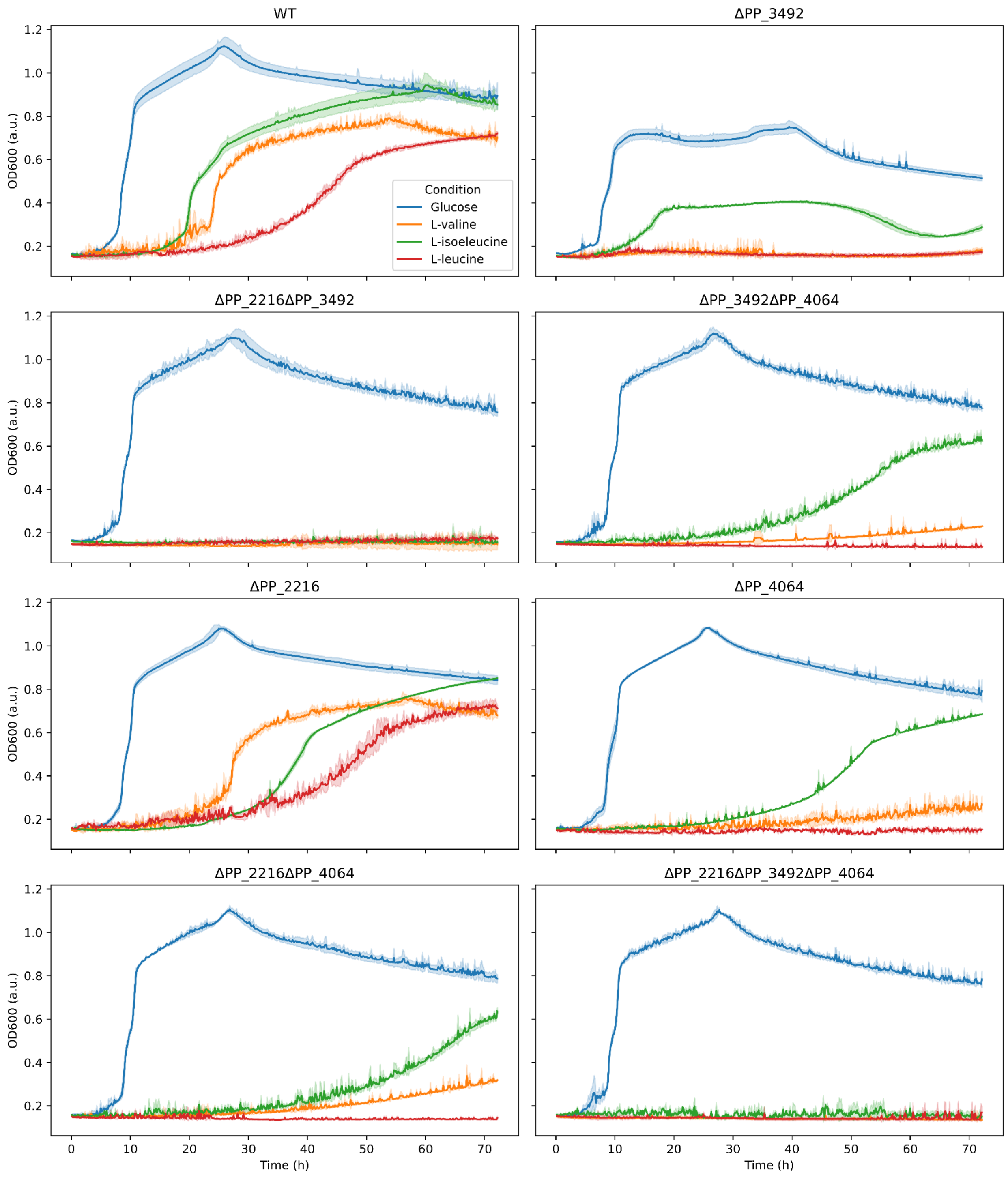

Supplementary Figure 1: Plate-based growth assay with acyl-CoA dehydrogenase mutants of *Pseudomonas putida*. The growth assay was conducted in mM9 minimal medium with glucose (blue), L-valine (yellow), L-isoleucine (green), or L-leucine (red) as the sole source of carbon. BarSeq analysis revealed that PP_2216 causes a fitness defect in growth on L-isoleucine (-4.4), PP_4064 results in fitness defects for growth on both L-valine (-2.0) and L-leucine (-3.5), and

PP_3492 causes a fitness defect for growth on L-valine (-2.1). WT: wild-type. Error bars represent the standard deviation of n = 3.

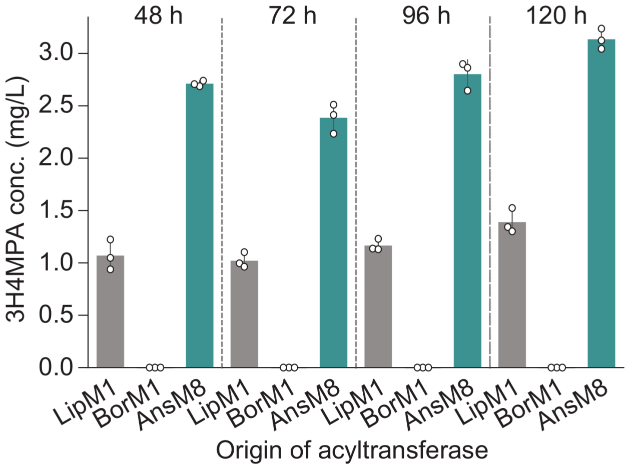

Supplementary Figure 2: Time course of 3-hydroxy-4-methylpentanoic acid (3H4MPA) production with acyl transferase-exchanged lipomycin polyketide synthase (LipPKS) mutants in *Pseudomonas putida*. The production of the polyketide 3H4MPA was achieved by condensing isobutyryl-CoA (ibCoA) and malonyl-CoA (mCoA). Error bars represent the standard deviation of n = 3.

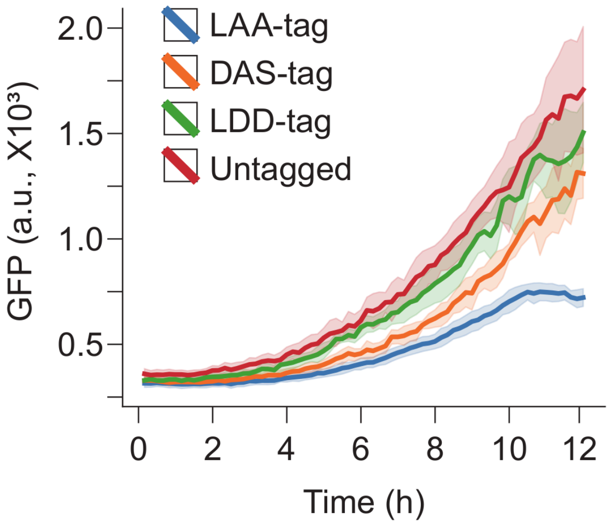

Supplementary Figure 3: Time course of ssrA-tagged green fluorescent protein (GFP) levels in *Pseudomonas putida.* Error bars represent the standard deviation of n = 3.

Bibliography

Bi, C., Su, P., Müller, J., Yeh, Y.-C., Chhabra, S.R., Beller, H.R., Singer, S.W., Hillson, N.J., 2013. Development of a broad-host synthetic biology toolbox for Ralstonia eutropha and its application to engineering hydrocarbon biofuel production. Microb. Cell Fact. 12, 107. https://doi.org/10.1186/1475-2859-12-107.

Boyer, H.W., Roulland-Dussoix, D., 1969. A complementation analysis of the restriction and modification of DNA in Escherichia coli. J. Mol. Biol. 41, 459–472. https://doi.org/10.1016/0022-2836(69)90288-5.

Choi, K.-H., Gaynor, J.B., White, K.G., Lopez, C., Bosio, C.M., Karkhoff-Schweizer, R.R., Schweizer, H.P., 2005. A Tn7-based broad-range bacterial cloning and expression system. Nat. Methods 2, 443–448. https://doi.org/10.1038/nmeth765.

Elmore, J.R., Dexter, G.N., Baldino, H., Huenemann, J.D., Francis, R., Peabody, G.L., Martinez-Baird, J., Riley, L.A., Simmons, T., Coleman-Derr, D., Guss, A.M., Egbert, R.G., 2023. High-throughput genetic engineering of nonmodel and undomesticated bacteria via iterative site-specific genome integration. Sci. Adv. 9, eade1285. https://doi.org/10.1126/sciadv.ade1285.

Figurski, D.H., Helinski, D.R., 1979. Replication of an origin-containing derivative of plasmid RK2 dependent on a plasmid function provided in trans. Proc Natl Acad Sci USA 76, 1648–1652. https://doi.org/10.1073/pnas.76.4.1648.

Incha, M.R., Thompson, M.G., Blake-Hedges, J.M., Liu, Y., Pearson, A.N., Schmidt, M., Gin, J.W., Petzold, C.J., Deutschbauer, A.M., Keasling, J.D., 2020. Leveraging host metabolism for bisdemethoxycurcumin production in Pseudomonas putida. Metab. Eng. Commun. 10, e00119. https://doi.org/10.1016/j.mec.2019.e00119.

Schmidt, M., Lee, N., Zhan, C., Roberts, J.B., Nava, A.A., Keiser, L.S., Vilchez, A.A., Chen, Y., Petzold, C.J., Haushalter, R.W., Blank, L.M., Keasling, J.D., 2023. Maximizing heterologous expression of engineered type I polyketide synthases: investigating codon optimization strategies. ACS Synth. Biol. 12, 3366–3380. https://doi.org/10.1021/acssynbio.3c00367.

Shanks, R.M.Q., Caiazza, N.C., Hinsa, S.M., Toutain, C.M., O'Toole, G.A., 2006. *Saccharomyces cerevisiae*-based molecular tool kit for manipulation of genes from gram-negative bacteria. Appl. Environ. Microbiol. 72, 5027–5036. https://doi.org/10.1128/AEM.00682-06.

Zobel, S., Benedetti, I., Eisenbach, L., de Lorenzo, V., Wierckx, N., Blank, L.M., 2015. Tn7-Based Device for Calibrated Heterologous Gene Expression in Pseudomonas putida. ACS Synth. Biol. 4, 1341–1351. https://doi.org/10.1021/acssynbio.5b00058.
